# Airway response to acute mechanical stress in a human bronchial model of stretch

**DOI:** 10.1186/cc10443

**Published:** 2011-09-13

**Authors:** Christophe Faisy, Francisco M Pinto, Morgan Le Guen, Emmanuel Naline, Stanislas Grassin Delyle, Edouard Sage, Maria-Luz Candenas, Philippe Devillier

**Affiliations:** 1 Research Unit UPRES EA220, University Versailles-Saint-Quentin, Hôpital Foch, 40 rue Worth, Suresnes 92150, France; 2 Medical Intensive Care Unit, European Georges Pompidou Hospital, 20 rue Leblanc, Paris Cedex 15, 75908, France; 3 Instituto de Investigaciones Quimicas, CSIC, Av. Americo Vespucio, S/N. Isla De La Cartuja, Sevilla, 41092, Spain; 4 Department of Thoracic Surgery, Hôpital Foch, 40 rue Worth, Suresnes, 92150, France

## Abstract

**Introduction:**

Lung inflation may have deleterious effects on the alveoli during mechanical ventilation. However, the consequences of stretch during excessive lung inflation on basal tone and responsiveness of human bronchi are unknown. This study was undertaken to devise an experimental model of acute mechanical stretch in isolated human bronchi and to investigate its effect on airway tone and responsiveness.

**Methods:**

Bronchi were removed from 48 thoracic surgery patients. After preparation and equilibration in an organ bath, bronchial rings were stretched for 5 min using a force (2.5 × basal tone) that corresponded to airway-inflation pressure > 30 cm H_2_O. The consequences of stretch were examined by using functional experiments, analysis of organ-bath fluid, and ribonucleic acid (RNA) isolation from tissue samples.

**Results:**

Following removal of the applied force the airways immediately developed an increase in basal tone (*P *< 0.0001 vs. paired controls) that was sustained and it did so without significantly increasing responsiveness to acetylcholine. The spontaneous tone was abolished with a Rho-kinase inhibitor and epithelium removal, a leukotriene antagonist or nitric oxide synthase inhibitors reduced it, whereas indomethacin, sensory nerve inhibitors or antagonists for muscarinic, endothelin and histamine receptors had no effect. Stretch enhanced leukotriene-E_4 _production during the immediate spontaneous contraction of human bronchi (*P *< 0.05). Moreover, stretch up-regulated the early mRNA expression of genes involved in wingless-type mouse mammary tumor virus integration-site family (WNT)-signaling and Rho-kinase pathways.

**Conclusions:**

Stretching human bronchi for only 5 min induces epithelial leukotriene release via nitric oxide synthase activation and provokes a myogenic response dependent on Rho-kinase and WNT-signaling pathways. From a clinical perspective, these findings highlight the response of human airway to acute mechanical stress during excessive pulmonary inflation.

## Introduction

In healthy subjects, air movement into and out of the lungs throughout respiration produces estimated pressure variations of 5 to 25 cm H_2_O, corresponding to functional residual capacity and total lung capacity, respectively. Airway inflation of the lungs induces mechanical strain and in turn causes either smooth muscle relaxation or contraction, which are mediated for the most part by airway epithelium and mechanosensors, such as slowly and rapidly adapting receptors and C-fiber receptors [1,2]. Excessive airway inflation leads to up-regulation of the genes encoding inflammatory protein expression and mediator secretion by airway cells [3]. Mechanical ventilation could enhance alteration of mechanical forces in the lungs of patients with asthma, emphysema or acute respiratory distress syndrome, thereby causing ventilator-associated lung injury. Damage to the epithelial cell lining of the airways and alveoli during high tidal volume ventilation have been extensively investigated but the effects of excessive pulmonary ventilation on airway basal tone and bronchial responsiveness are not well known in humans [2,4-6].

Mechanical strain imposed on airway cells induce a cascade of signaling events, primarily mediated by the macromolecular protein complexes associated with the transmembrane integrins that transduce the external forces from the extracellular matrix to the actin cytoskeleton, resulting in the production of various mediators, cytokines, and growth factors, coupled with gene activation [6,7]. The mechanotransduction induced by integrin activation involves a variety of intracellular-signaling pathways mediated by protein kinases, eicosanoids, nitric oxide synthases (NOS), reactive oxygen species, proinflammatory cytokines and stretch-activated channels [5,8-12]. Furthermore, stretch-induced mechanotransduction is dependent on immediate early gene activation, especially the overexpressed cysteine-rich 61/connective tissue growth factor/nephroblastoma (CCN) family [3,13]. WNT (wingless-type mouse mammary tumor virus integration-site family), a large family of secreted glycoproteins with highly conserved cysteine residues, may also be involved in cytoskeletal reorganization after airway stretching [14]. However, the pathways involved in the stretch-induced mechanotransduction in freshly isolated human bronchus remain unknown and results extrapolated from cell-culture systems in either static or under cyclic strain conditions have limitations. Similarly, the myogenic response to stretch is not well understood for intact human bronchi. Our study was conducted to establish an experimental model of static acute mechanical stretch that corresponded to airway hyperinflation in isolated human bronchi to investigate the effect of stretch on airway tone and responsiveness.

## Materials and methods

The study was approved by our local Ethics Committees (Comité de Protection des Personnes se Prêtant à la Recherche Biomédicale de Versailles, France, and Consejo Superior de Investigaciones Científicas, Madrid, Spain) and patients gave informed consent before scheduled surgery. Bronchi were surgically removed from 48 patients with lung cancer (35 men, 13 women, mean age 64 ± 9 years; all patients were smokers or ex-smokers).

### Bronchus preparations

Just after resection, segments of mid-sized (inner diameter 1.5 to 3 mm) human bronchi were excised as far as possible from the malignant lesion and the absence of tumoral infiltration was retrospectively established in all bronchi. After removal of adhering lung parenchyma and connective tissue, bronchi were washed in oxygenated Krebs-Henseleit solution (composition mM: NaCl 119, KCl 4.7, CaCl_2 _2.5, KH_2_PO_4 _1.2, NaHCO_3 _25 and glucose 11.7). Bronchial rings of similar lengths were prepared and then suspended on hooks in a 5-ml organ bath containing Krebs-Henseleit solution, gassed with 95% O_2_, 5% CO_2 _and maintained at 37°C. Each preparation was connected to a force displacement transducer and isometric tension changes were recorded on a polygraph. Rings were equilibrated for at least 60 minutes with changes of fresh Krebs-Henseleit solution every 10 minutes during the first 30 minutes of the equilibration period. Bronchi were suspended with an initial tension of 1 g [15]. When required, the epithelium was removed before suspension in the organ bath as previously described [16]. After experiments, rings that had been patted dry were weighed.

### Mechanical stress

After tissue equilibration, rings were stretched for five minutes by increasing tension by 2, 2.5 or 3-fold. A strain of 2-fold or more than the basal tone was chosen because it corresponds to an airway-inflation pressure more than 30 cmH_2_O in mid-sized human and porcine bronchi [17]. Once post-stretch basal tone was re-stabilized, concentration-response curves to acetylcholine (Ach; 0.1 μM to 10 mM) were then obtained. Contractile responses are expressed as g/basal tone recorded immediately before obtaining concentration-response curves to ACh. Emax (g) represents the maximal contraction induced by 10 mM ACh. ΔEmax represents the difference between Emax obtained with the stretched bronchi and Emax obtained with their paired controls. ACh potency (-log EC_50_) was derived graphically from the log_10 _concentration-effect curves and defined as the negative log_10 _of the ACh concentration achieving 50% of the maximal 10 mM ACh effect. Δ(-log EC_50_) represents the difference between -log EC_50 _obtained with the stretched bronchi and -log EC_50 _obtained with the paired control human bronchi.

### Functional study

To investigate the different signaling pathways potentially implicated in the stretch-induced response, experiments were run in parallel (control and pretreated groups) in the absence or presence of drugs added immediately to the organ bath after the last change of fresh Krebs-Henseleit solution during the equilibration period: 1) a cyclooxygenase (COX) inhibitor: indomethacin (0.1 μM); 2) a leukotriene Cyst-LT_1 _receptor antagonist: MK476 (0.1 μM); 3) a nonspecific NOS inhibitor at high concentration and specific NOS_3 _inhibitor at low concentration = L-nitroarginine methyl ester (L-NAME, 1 mM and 1 μM, respectively) [11,18,19]; 4) a selective inhibitor of inducible NOS_2_: 1400 W (1 mM) [11]; 5) a selective inhibitor of constitutive NOS_1_: N^ω^-propyl-L-arginine (5 mM) [11]; 6) a mixture of the tachykinin NK_1_-, NK_2_-, and NK_3_-receptor antagonists: SR 140333 (0.1 μM), SR 48968 (0.1 μM), SR 142801 (0.1 μM) [20]; 7) a histamine H_1_-receptor antagonist: mepyramine (0.1 μM); 8) a mixture of the selective endothelin ET_A_- and ET_B_-receptor antagonists: BQ 123 (0.1 μM) and BQ 788 (0.1 μM); 9) a muscarinic ACh-receptor antagonist: atropine (0.1 μM); 10) a nonspecific blocker of acid-sensing ion and stretch-activated channels: gadolinium (Gd^3+^, 0.1 mM) [21,22]; 11) a selective Rho-kinase (ROCK1 and ROCK2) inhibitor: Y27632 (0.1 μM) [23,24]. ACh, indomethacin, L-NAME, mepyramine, BQ 123, BQ 788, atropine and gadolinium were purchased from Sigma-Aldrich (St. Louis, MO, USA). MK476 came from Merck Sharp & Chibret (Paris, France). 1400 W and N^ω^-propyl-L-arginine were purchased from Cayman Chemicals (Ann Arbor, MI, USA). SR 140333, SR 142801 and SR 48968 were provided by Sanofi-Aventis Research (Montpellier, France). Y27632 was purchased from Alexis Biochemicals (San Diego, CA, USA). All drugs were dissolved in distilled water except for indomethacin, 1400 W and N^ω^-propyl-L-arginine which were dissolved in pure ethanol and then diluted in Krebs solution. The final ethanol concentration (0.03%) did not alter airway tone or contractility.

### Analysis of organ-bath fluid

Concentrations of prostaglandins (PG) and leukotrienes (LT) in the organ bath were measured immediately before and 15 minutes after stretch by sampling 250 μl of organ-bath fluid each time from stretched bronchi and their paired controls. Individual samples were assayed using specific ELISA kits for the stable excretory LTE_4_, PG screening, and PGE_2 _(all from Cayman Chemical Company, Ann Arbor, MI, USA). Optical density was read on the microplate autoreader (MRX II, Dynex Technologies, Chantilly, VA, USA). The mean net optical density of the standards was plotted and the individual sample concentrations were read from the standard curves. For each assay, all samples were analyzed on the same day in a blinded fashion. Results were expressed as the means of duplicate samples. Stretch-induced production of LTE_4 _and PG are expressed in pg/mg of bronchial tissue and corresponds to the difference of the amount (concentration × volume) of LTE_4 _or PG excreted into the organ bath immediately before and 15 minutes after stretching.

### RNA isolation and RT-PCR array

To isolate RNA, paired bronchial rings were immediately immersed in RNAlater (Sigma, St. Louis, MO, USA) after preparation (control H0) or were stretched (5 minutes) or non-stretched and then stabilized for three hours in an organ bath before immersion in RNA later. No concentration-response curves to ACh were obtained. Bronchi were then stored at -80°C until further use. Total RNA was isolated with the RNeasy Fibrous Tissue kit (Qiagen, Venlo, The Netherlands) and quantified spectrophotometrically at 260 nm. Genomic DNA contamination was removed and retrotranscription performed with RT first-strand kit (SABiosciences, Frederick, MD, USA). Gene expression in template cDNAs was characterized with the Human Signal Transduction and the human WNT-signaling PathwayFinder RT^2 ^Profiler PCR Arrays (SABiosciences, Frederick, MD, USA). The human signal transduction finder pathway PCR array was used to investigate the pathways involved in the stretch-induced response. From the results obtained in these experiments, we carried out further investigations using WNT-signaling pathway PCR array, which analyses the expression of a focused panel of genes related to the different pathways regulated by *WNT*. These real-time quantitative PCR arrays generate expression patterns of different genes products involved in inflammation, migration-adhesion, growth-differentiation, apoptosis, WNT-mediated signal transduction, and five housekeeping genes to normalize the PCR-array data. The kit also contains a control for genomic DNA contamination, three replicate RT controls and three positive replicate controls of the PCR reaction. Real-time PCR was performed on a Bio-Rad iCycler iQ real-time detection apparatus (Bio-Rad Laboratories, Hercules, CA, USA) using a RT SYBR Green/Fluorescein qPCR Master Mix purchased from SABiosciences (Frederick, MD, USA). After a hot start (10 minutes at 95°C), the parameters used for PCR amplification were: 15 seconds at 95°C, 30 seconds at 60°C, and 30 seconds at 72°C for 45 cycles, and fluorescence was measured after amplification. At the end of each PCR run, an amplification plot was generated for each DNA sample. From each of these plots, the iCycler software calculated the threshold cycle (C_T_) value for each gene on each PCR array. Quantitative real-time PCR values are expressed as the fold change of each target-gene expression compared with the mean mRNA expression of the five housekeeping genes in each sample. Gene-expression fold changes were calculated according to the ΔΔC_T _method using the Web-based software provided by SuperArray Biosciences Corp (Frederick, MD, USA). mRNA expression levels are shown as the ratio of stretched or non-stretched bronchi/paired control H0. A positive or negative value indicates respective gene up- or down-regulation.

### Statistics

Values are presented as means ± standard error of the mean. The results were analyzed using Student's *t *test for paired data and repeated measures analysis of variance with Bonferroni adjustments for multiple comparisons (StatView 5.0, SAS Institute, Cary, NC, USA). A *P *value of less than 0.05 was considered statistically significant.

## Results

### Mechanical stress

**Figure 1:**
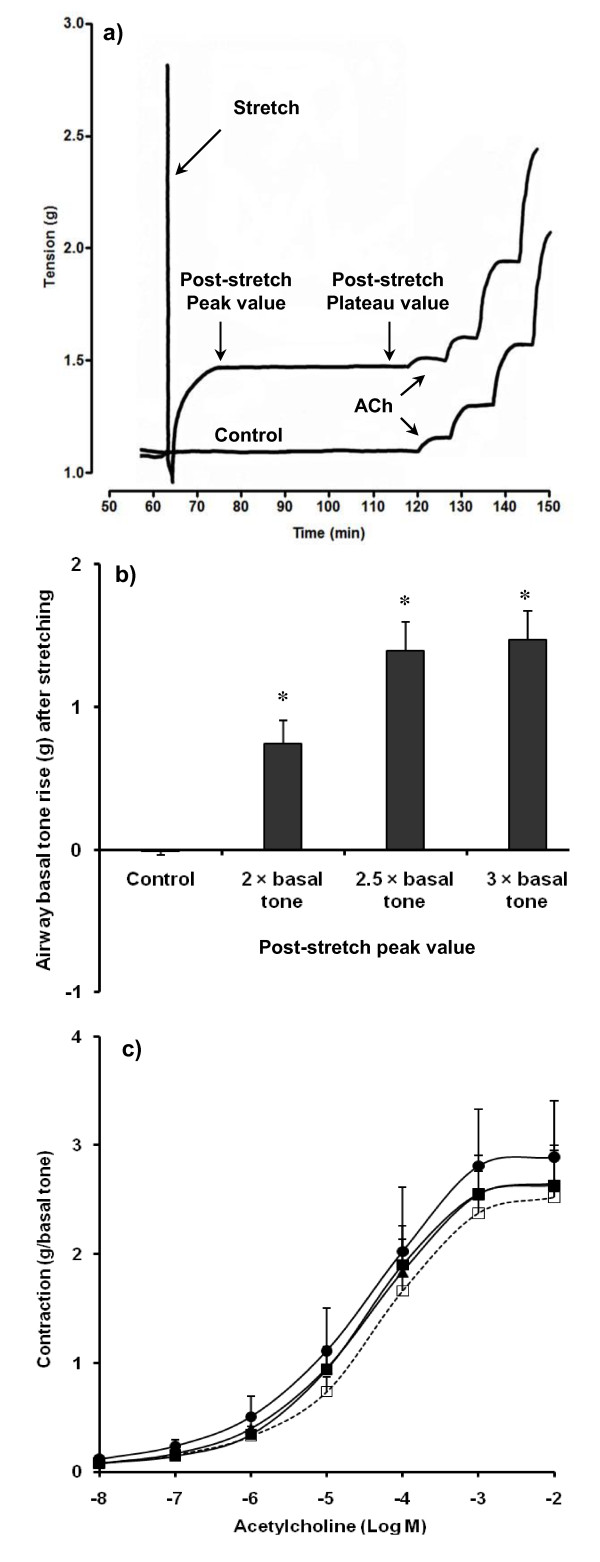
**Effect of mechanical stress on airway tone and responsiveness**. **(a) **Basal tone and responsiveness to acetylcholine (ACh) in single control and stretched human bronchial rings. After tissue equilibration in an organ bath for 60 minutes, acute mechanical stretch (5 minutes, 2.5 × basal tone) provokes a quick rise of basal tone (immediate contraction corresponding to post-stretch peak) followed by a prolonged plateau (sustained contraction). **(b) **Post-stretch peak is strain-dependent with a maximal response for a tension corresponding to 2.5-fold resting basal tone in 11 human bronchial rings. **(c) **Responsiveness to ACh is not significantly altered by stretching in the same 11 paired bronchial rings. Filled squares, stretch 2 × basal tone; filled triangles, stretch 2.5 × basal tone; filled circles, stretch 3 × basal tone; open squares, paired controls. Values are means ± standard error of the mean. *P < 0.001 vs. paired control.

In preliminary experiments (*n *= 11), following a five minute stretch of the tissue tone spontaneously increased and reached a sustained plateau in 11 ± 1 minutes (Figure 1a). The magnitude of the effect was also dependent of the applied force (Figure 1b). As spontaneous tone development was greatest following the stretch protocol which increased tension to 2.5 times passive tone, this amplitude of stretch was chosen for further studies. Basal tone was recorded when it reached a peak after stretching and when re-stabilization (plateau) was maintained for at least 30 minutes, immediately before obtaining concentration-response curves to ACh. Moreover, stretch did not significantly modify the ACh-induced contraction of human bronchi (Figure 1c).

Mean patted-dry weight of stretched bronchi was comparable with that of their paired controls (37.3 ± 3.6 vs. 38.3 ± 3.4 mg, *P *= 0.57, *n *= 48). Stretch (5 minutes, 2.5 × basal tone) induced a basal tone rise of 1.22 ± 0.08 g at peak and 1.16 ± 0.08 g at plateau (all *P *< 0.0001 vs. paired control, *n *= 48). ΔEmax ACh was 0.22 ± 0.20 g and Δ(-log EC_50_) ACh was 0.04 log units between stretched bronchi and their paired controls (all > 0.05, *n *= 48).

### Functional study

Epithelium removal, leukotriene Cyst-LT_1_-receptor blockage and selective inhibition of NOS_1,2,3 _resulted in a significantly reduced rise in stretch-induced spontaneous tone (Table 1). Y27632 inhibition of Rho-kinase pathway abolished the stretch-effect on basal tone. In contrast, blocking/inhibiting NK_1,2,3_, histamine, endothelin or muscarinic receptors, acid-sensing ion or stretch-activated channels or COX had no effect on the stretch-induced basal tone rise. Only gadolinium and BQ 123 + BQ 788 affected ACh potency or maximal efficacy by significantly increasing the ΔEmax ACh recorded after stretching (Table 2).

**Table 1:** Effects of pretreatment on the rise of airway basal tone (g) in human bronchi at peak or plateau post-stretch (5 minutes, 2

		Post-stretch peak		Post-stretch plateau	
Pretreatment	n	Control	Pretreated	Control	Pretreated
Epithelium removal	12	1.43 ± 0.21	0.93 ± 0.15^*b*^	1.38 ± 0.22	0.89 ± 0.16^*b*^
MK476 (0.1 μM)	13	1.47 ± 0.19	0.89 ± 0.18^*a*^	1.45 ± 0.19	0.61 ± 0.18^*b*^
Indomethacin (1 μM)	9	1.25 ± 0.23	1.29 ± 0.23	1.21 ± 0.24	1.21 ± 0.21
1400 W (1 mM)	11	1.19 ± 0.11	0.89 ± 0.14^*a*^	1.13 ± 0.11	0.79 ± 0.16^*a*^
L-NAME (1 μM)	9	1.16 ± 0.12	0.97 ± 0.29	1.09 ± 0.11	0.91 ± 0.29
L-NAME (1 mM)	13	1.37 ± 0.20	0.86 ± 0.16^*b*^	1.31 ± 0.22	0.80 ± 0.16^*b*^
N^ω^-propyl-L-arginine (5 mM)	11	1.19 ± 0.11	0.83 ± 0.09^*a*^	1.13 ± 0.11	0.84 ± 0.10^*a*^
SR 140333 + SR 48968 + SR 142801 (0.1 μM each)	9	1.01 ± 0.12	0.90 ± 0.16	0.96 ± 0.12	0.92 ± 0.17
BQ 123 + BQ 788 (0.1 μM each)	6	1.08 ± 0.16	1.20 ± 0.17	1.01 ± 0.14	1.17 ± 0.24
Atropine (0.1 μM)	9	1.23 ± 0.20	1.02 ± 0.22	1.16 ± 0.20	1.05 ± 0.22
Gadolinium (0.1 mM)	7	1.12 ± 0.11	0.98 ± 0.14	1.05 ± 0.12	0.95 ± 0.15
Mepyramine (1 μM)	9	1.15 ± 0.24	1.33 ± 0.20	1.11 ± 0.24	1.24 ± 0.23
Y27632 (1 μM)	7	1.12 ± 0.11	0.15 ± 0.07^*c*^	1.05 ± 0.12	0.11 ± 0.05^*c*^

Values are means ± standard error of the mean. *^a^P *< 0.05; *^b^P *< 0.01; *^c^P *< 0.001 vs. paired control. 1400 W, selective inhibitor of inducible nitric oxide synthase; BQ 123, selective endothelin ET_A_-receptor antagonist; BQ 788, selective endothelin ET_B_-receptor antagonist; L-NAME, L-nitroarginine methyl ester; MK476, leukotriene Cyst-LT_1 _receptor antagonist; SR 140333, tachykinin NK_1_-receptor antagonist; SR 48968, tachykinin NK_2_-receptor antagonist; SR 142801, tachykinin NK_3_-receptor antagonist; Y27632, selective Rho-kinase (ROCK1 and ROCK2) inhibitor.

**Table 2:** Effects of pretreatments on airway responsiveness in human bronchi post-stretch (5 minutes, 2

		Δ(-log EC_50_) ACh (log unit)		ΔEmax ACh (g)	
Pretreatment	n	Control	Pretreated	Control	Pretreated
Epithelium removal	12	0.31 ± 0.12	-0.02 ± 0.14	0.28 ± 0.23	-0.06 ± 0.19
MK476 (0.1 μM)	13	0.12 ± 0.22	0.10 ± 0.24	1.03 ± 0.57	-0.30 ± 0.44
Indomethacin (1 μM)	9	0.08 ± 0.30	0.77 ± 0.39	0.74 ± 0.86	1.29 ± 0.59
1400 W (1 mM)	11	0.02 ± 0.18	-0.20 ± 0.28	0.22 ± 0.33	-0.02 ± 0.26
L-NAME (1 μM)	9	0.16 ± 0.17	0.24 ± 0.11	0.45 ± 0.24	-0.04 ± 0.25
L-NAME (1 mM)	13	0.15 ± 0.25	-0.08 ± 0.13	0.11 ± 0.21	-0.16 ± 0.22
N^ω^-propyl-L-arginine (5 mM)	11	0.02 ± 0.18	0.34 ± 0.20	0.22 ± 0.33	0.14 ± 0.61
SR 140333 + SR 48968 + SR 142801 (0.1 μM each)	9	0.30 ± 0.16	0.11 ± 0.17	0.02 ± 0.09	0.14 ± 0.42
BQ 123 + BQ 788 (0.1 μM each)	6	0.17 ± 0.19	0.72 ± 0.20	-0.35 ± 0.22	1.12 ± 0.32^*a*^
Gadolinium (0.1 mM)	7	0.24 ± 0.17	0.47 ± 0.15	-0.16 ± 0.27	1.34 ± 0.42^*b*^
Mepyramine (1 μM)	9	-0.03 ± 0.36	-0.09 ± 0.24	1.45 ± 0.81	0.58 ± 0.46
Y27632 (1 μM)	7	0.24 ± 0.17	-0.02 ± 0.10	-0.16 ± 0.27	0.05 ± 0.31

Values are means ± standard error of the mean. ^*a*^*P *< 0.01; *^b^P *< 0.001 vs. paired control. 1400 W, selective inhibitor of inducible nitric oxide synthase; Ach, acetylcholine; BQ 123, selective endothelin ET_A_-receptor antagonist; BQ 788, selective endothelin ET_B_-receptor antagonist; L-NAME, L-nitroarginine methyl ester; MK476, leukotriene Cyst-LT_1 _receptor antagonist; SR 140333, tachykinin NK_1_-receptor antagonist; SR 48968, tachykinin NK_2_-receptor antagonist; SR 142801, tachykinin NK_3_-receptor antagonist; Y27632, selective Rho-kinase (ROCK1 and ROCK2) inhibitor.

### ELISA analysis of organ-bath fluid

**Figure 2:**
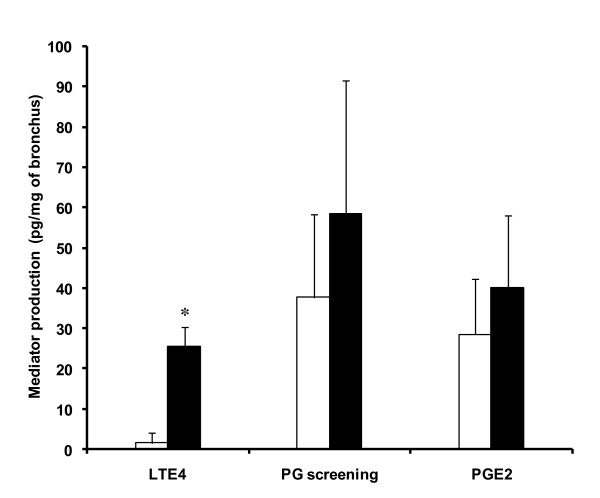
**ELISA analysis of organ-bath fluid**. Effect of stretch (5 minutes, 2.5 × basal tone) on the production of leukotriene E_4 _(LTE_4_), prostaglandin screening (PG), and prostaglandin E_2 _(PGE_2_) in an organ bath assessed by ELISA during the immediate contraction following stretch (Figure 1) in 14 human bronchi (controls, open bars). Stretch (filled bars) induced a higher stable excretory LTE_4 _production. Values are means ± standard error of the mean. *P < 0.05 vs. paired control.

Stretching human bronchi significantly increased LTE_4 _release compared with paired controls (Figure 2). Neither PG nor PGE_2 _release were significantly increased.

### RT-PCR

**Figure 3:**
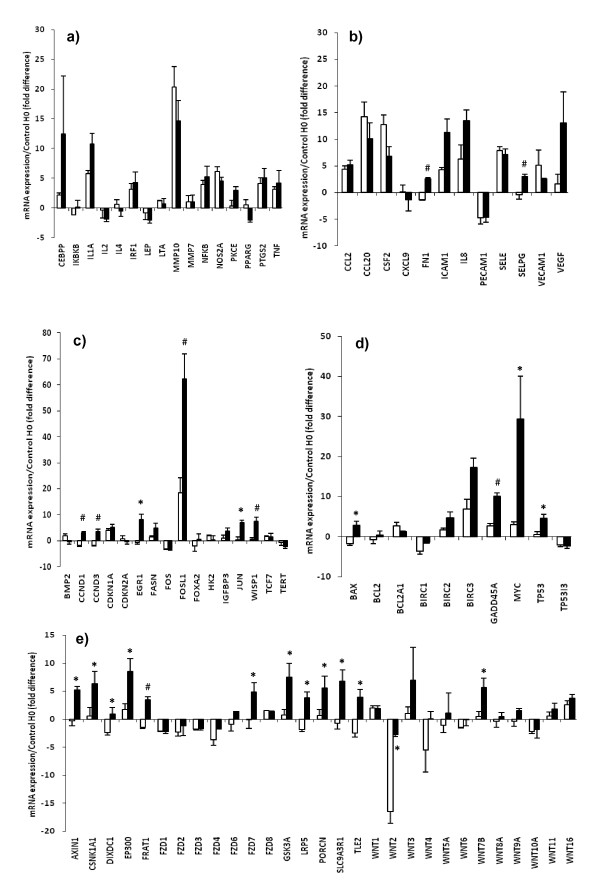
**RT-PCR array**. Effect of stretch (5 minutes, 2.5 × basal tone) on the early mRNA expression (3 hours after stretching) of genes implicated in **(a) **cell inflammation, **(b) **cell migration-adhesion, **(c) **cell growth-differentiation, **(d) **cell apoptosis, and **(e) **Wnt-mediated signal transduction. mRNA expression represents the ratio of experimental value/H0 value (fold difference). Filled bars, stretch; open bars, paired controls. Values are means ± standard error of the mean (*n *= 9). *P < 0.01; **^#^***P *< 0.001 vs. paired control.

mRNA levels of the five housekeeping genes were unaffected by the interval between bronchus extraction on H0 onset and suspension in organ bath (Table 3). Control gene mRNA levels in stretched tissues were comparable with those observed in non-stretched tissues. Compared with paired controls, stretch did not significantly change mRNA expression of genes implicated in inflammation of human bronchi (Figure 3a). Stretch led to significantly higher mRNA levels of: *FN1, SELPG, CCND1, CCND3, EGR1, FOSL1, JUN, WISP1, BAX, GADD45A, MYC*, and *TP53I3 *(Figures 3b to 3d). Moreover, stretch up-regulated mRNA expression of various genes involved in the WNT-signaling pathway, especially *WNT7B *(Figure 3e). Stretch also abolished the increased *WNT2 *repression that what observed in paired control bronchi maintained in an organ bath.

**Table 3:** ΔmRNA for the housekeeping genes (*ACTB, B2M, GAPDH, HPRT1*, and *RPL13A*) in human bronchi after suspension in an organ bath with (5 minutes, 2

	No stretch		Stretch	
*Gene*	Paired rings	ΔmRNA vs. Control H0	Paired rings	ΔmRNA vs. Control H0
*ACTB*	9	0.11 ± 0.58	9	0.01 ± 0.62
*B2M*	9	-0.40 ± 0.46	9	-0.40 ± 0.45
*GAPDH*	9	1.18 ± 0.42	9	-0.88 ± 0.39
*HPRT1*	9	0.14 ± 0.59	9	-0.31 ± 0.61
*RPL13A*	9	-0.07 ± 0.52	9	0.47 ± 0.50

After normalization to the mean of the five housekeeping genes, ΔmRNA for each target gene was calculated as the fold mRNA-value change of each non-stretched or stretched ring relative to the mRNA value in its paired control H0. Values are mean ± standard error of the mean. No significant differences in expression of house-keeping genes were observed. *ACTB*, β-actin; *B2M*, β_2_-microglobulin; *GAPDH*, glyceraldhyde-3-phosphate dehydrogenase; *HPRT1*, hypoxantine phosphoribosyltransferase 1; *RPL13A*, ribosomal protein L13α.

## Discussion

The results of this study demonstrated that stretching human isolated bronchi for only five minutes while using a force that corresponded to an inflation pressure of more than 30 cmH_2_O increased basal tone, but did not increase tissue responsiveness to ACh. The stretch-induced increase in basal tone was Rho-kinase dependent and in part bronchial epithelium dependent. Some mediators were also implicated, such as leukotrienes and reactive oxygen species, which may have been released from the epithelium. Interestingly, the five minute stretch resulted in the up-regulation of the WNT-signaling, which is known to interact with the Rho-kinase pathway [25]. Activation of these intracellular signaling-pathways could have clinical relevance in mechanically ventilated patients, especially those with obstructive pulmonary diseases. Indeed, lung inflation has been shown to alter the airway smooth muscle functions [26]. In normal adults with induced bronchoconstriction, a deep inspiration results in bronchodilatation. For unclear reasons, this is impaired in patients with asthma and chronic obstructive pulmonary disease [27]. Moreover, a large body of work suggests that imposed tidal fluctuations of airway smooth muscle length relax isolated airway smooth muscle strips [26].

In mechanically ventilated rabbits, the bronchodilating effect of changes in lung volume is caused by the direct effect of stretch on force generation by the airway smooth muscle [28]. Lung inflation strains airway smooth muscle and causes a dynamic modulation of cytoskeletal filament organization such as rearrangement of actin and myosin filaments, detachment and attachment of actin filaments and plaques, and polymerization and depolymerization of these filaments [26,29]. In oscillatory experiments, it is the very first tidal oscillation that provokes most of any force and stiffness reduction in cells or tissues [29]. A single transient stretch may acutely fluidize airway smooth muscle which is normally solid, leading to perturbed equilibrium of myosin binding and alteration of airway smooth muscle mechanical properties (the cell becomes much less stiff and much more viscous) [30]. When stretch is stopped, the cell resolidifies but the muscle reshortens to a new biophysical state different from the prior under identical loading conditions [30]. Moreover, the relaxing effect persists even if stretch oscillation amplitude is reduced [28]. Herein, the absence of hyper-responsiveness following stretch could be explained by the stretch-induced perturbation of myosin binding [29,30]. In addition, we found epithelial removal failed to enhance responsiveness to ACh after stretch, suggesting excessive mechanical strain also alters the epithelial modulation of airway smooth muscle contraction. However, the hypothesis that force fluctuations on airway smooth muscle can diminish airway tone and responsiveness has been recently challenged by LaPrad and colleagues in healthy intact airway [31], questioning the role of cytoskeleton fluidization on airway responsiveness [32].

Consistent with our results using whole intact human tissue, Hernandez and colleagues showed that bovine airway segments also responded similarly to stretch, but only following pretreatment with selected contractile agonists [20]. This strain-induced increase in basal tone could reflect length adaptation of the airway smooth muscle: next a stretch and return, the cytoskeleton fluidizes softening, which is then followed by an increase in attachment of actin filaments and increase in tone [26,29]. Indeed, basal tone is critically influenced by the length at which the airway smooth muscle is adapted [33]. After priming the contractile apparatus induced by pretension or contractile agonists such as ACh, quick stretch or very first force oscillations might cause contractile apparatus rearrangements, with many myosin heads interacting with actin filaments, leading to regeneration of a force exceeding prestretch levels [5,20,34,35]. The strain-stiffening behavior of human airway smooth-muscle cells observed in response to sustained stretch or reconstituted cross-linked actin gels could also explain in part the increase in basal tone [29,36]. However, the myogenic response to stretch did not entirely explain the effects of various inhibitors or receptor antagonists on the stretch-induced spontaneous airway-smooth muscle contraction observed previously and herein [9,12,19,20,37].

In addition to direct mechanical injury, lung injury may be also caused by cell-mediated inflammation and soluble mediators such as TNF-α, IL-1β, IL-6, IL-8 and substance P [38-44]. Cytokine release can be related to the degree of lung stretch and inhibition of cytokine actions decreases stretch-induced injury [45,46]. However, as suggested by the present study, the mechanisms of stretch-induced injury in intact human bronchi appear to be more complex involving neural, mechanical, and mediator effects. The mechanotransduction involved in the force generation in the immediate response to stretching airways should involve pathways with short activation times, for example, such as mediator release or neurogenic mechanisms or cellular Ca^2+ ^influx via membrane depolarization caused by the stimulation of acid-sensing ion and stretch-activated channels [12,20,47]. Herein, we showed that the rapid rise in basal tone following stretch of human bronchi was abolished in the presence of a Rho-kinase inhibitor or attenuated after epithelium removal, blocking of the Cys-LT_1 _receptor or inhibition of NOS_1,2,3_. These results differ from those reported previously when using precontracted tissue from other species [12,20,48]. Differences among bovine and rodent species models of airway response to stretch have been reported previously [12,20,48]. Therefore, the discrepancies between our results and those from animal models are likely to be due to interspecies differences rather than differences among experimental protocols. For example, in the airways of guinea pigs, basal tone is dependent on COX products and epithelium, and stretch-induced spontaneous contraction is independent of LT [12,48]. Conversely, the intrinsic tone of human airways results from a balance of contractile LT and, to a lesser extent, histamine and bronchodilating prostanoids, like PGE_2 _[15,48,49]. Stretch modulates prostanoid synthesis by inhibiting COX-1 and activating COX-2, probably via stimulation of the inducible Ca^2+^-independent NOS_2 _and production of reactive oxygen species in airway epithelial cells [4,8,10-12]. It was also shown that airway epithelial cells could produce LT during inflammatory conditions or mechanical stretch [10,50]. Moreover, lung epithelial response to stretch appears to be extremely rapid and sensitive [4]. In accordance with these observations, our results confirmed the role of bronchial epithelium in enhancing the myogenic response to stretch and in particular the stretch-induced imbalance between prostanoid and LT production in human bronchi. However, mechanical stretch may induce LT release from others cells like bronchial smooth muscle and further investigations would examine stretch-induced prostanoid release in epithelium-denuded bronchial tissues. Taken together, these findings suggest that the immediate rise in basal tone following stretch of human bronchi involves increased epithelial LT production due to activation of Ca^2+^-independent inducible NOS_2_. In addition, the Rho-kinase inhibitor Y27632 abolishes the Ca^2+^-independent delayed contraction of airway smooth muscle by modulating COX-2 and NOS_2 _activation [12,24,29]. In isolated guinea-pig airways, stretch caused delayed contraction attributable to Ca^2+ ^influx into the muscle cells mediated by stretch-activated channel stimulation [12]. In rats exposed to injurious high airway pressure, substance P, which acts on the NK-1 and transient receptor potential ion channels, is implicated in ventilator-induced lung injury [43]. In our study gadolinium and the tachykinin NK_1_-, NK_2_-, and NK_3_-receptor antagonists had no significant effect on either the immediate or sustained phase of spontaneous basal tone provoked by stretch. Therefore, we cannot confirm that stretch-activated channels or substance P are involved in airway response to acute mechanical stress in intact isolated human bronchi.

In rodents, cyclical stretch induced by high tidal volume ventilation quickly increased transcription of several genes that regulate transcription like *EGR1, GADD45A *or *JUN*, whereas the induction of *TNF *mRNA expression was not significantly elevated, consistent with our results [3,39,51]. Mechanical stress also provoked vascular distension in rat trachea that may lead to activation of endothelial cells causing expression of P-selectin and endothelin-1 [40,52]. Interestingly, we showed herein that P-selectin ligand gene expression was increased by stretching human bronchi, whereas ET_A_- and ET_B_-receptor antagonists did not reduce airway response to stretch suggesting that endothelin-1 is not implicated in this phenomenon in human airways. The mechanotransduction leading to mechanical regulation of gene expression in contractile tissues has principally been investigated in bladder smooth muscle cells, where the Rho-kinase pathway seems to play a major role in the stretch-induced modulation of CCN-family genes [13,53,54], but it is not well known in human bronchi. To elucidate the signal transduction pathways that are activated in human bronchi following five minutes of stretch, we used the human signal transduction pathway finder PCR array, which analyses the expression of 84 genes representative of 18 different signal transduction pathways. The results of this assay showed that changes in early gene expression provoked by mechanical stretch involved genes implicated in cell proliferation-apoptosis or migration-adhesion but not cell inflammation in isolated human bronchi. Notably, we observed that stretching up-regulated the mRNA of genes implied in the WNT-signaling pathways. The *WNT *gene family includes 19 members encoding glycoproteins known as WNTs, which can activate two distinct signaling pathways (canonical and noncanonical), responsible for several cellular processes, including cell movement and polarity, proliferation and differentiation [14,25,55,56]. Our experiments with the human WNT-signaling pathway finder PCR array confirm that stretch modulates the mRNA levels of different genes of the WNT-signaling pathway, particularly *WNT7B *and *WNT2 *expression. WNT7b and WNT2 are expressed in the distal mesenchyme and in airway epithelium, and act via the seven membrane-spanning frizzled Wnt receptor (FZD) cell-surface receptors [14,25]. In addition, WNT7b activates the canonical but not the noncanonical pathway and exerts autocrine-signaling activity on airway epithelial cells [14]. Depending on the cellular context, WNTs stimulate the canonical signaling pathway via FZD receptors and nuclear translocation of β-cathenin, thereby up-regulating genes such as *WISP1, MYC, CCND1, CCND3, CSNK1N1, DIXDC1, EP300, FDZ7 *or *PORCN *[14,25], or activate the noncanonical signaling pathway through the Fzd receptors and ROR2/RYK coreceptors as suggested by the up-regulation of *JUN, FOSL1*, and *FRAT1 *observed herein. It is also known that WNT-signaling might induce cytoskeletal reorganization via up-regulation of *MYC, CCND1, CCND3 *[25,57], or via the activation of Rho-mitogen-activated protein kinase pathways elicited by the up-regulation of *GADD45A, SLC9A3R1 *and *TP53 *[58], as suggested by our results. Nevertheless, further investigations will be needed to confirm the functional role of the stretch-induced changes in mRNA expression, especially *WNT*.

## Conclusions

Collectively, our results indicate that the immediate component of myogenic response to stretch for human bronchi is mediated mostly by epithelial LT release via NOS activation and that the sustained component is dependent on Rho-kinase and WNT-signaling pathways. Moreover, the role of the bronchial epithelium in enhancing the myogenic response to stretch in our study illustrates the airway cells interactions during excessive pulmonary inflation. In this way, freshly human isolated bronchus represents a relevant tool to test the stretch-effects on complex networks of multiple airway cells. From a clinical perspective, our experimental model highlights the response of human airway to acute mechanical stress during excessive pulmonary inflation. Future work based on the results of the present study will be needed to determine the impact of long-term cyclic stretch on human airway mechanics, especially in mechanically ventilated patients with obstructive pulmonary diseases.

## Key messages

• The consequences of mechanical stress during excessive pulmonary ventilation on human bronchi are unknown.

• Airway response to acute mechanical stretch (using a force that corresponded to airway-inflation pressure > 30 cmH_2_O) in isolated human bronchi involves LT release, NOS activation and Rho-kinase and WNT-signaling pathways.

• This experimental model could help to appraise the impact of excessive pulmonary ventilation on human airway mechanics, especially in mechanically ventilated patients with obstructive pulmonary diseases.

## Abbreviations

ACh: acetylcholine; CCN: cysteine-rich 61/connective tissue growth factor/nephroblastoma; COX: cyclooxygenase; ELISA: enzyme-linked immunosorbent assay; FZD: frizzled Wnt receptor; IL: interleukin; L-NAME: L-nitroarginine methyl ester; LT: leukotriene; NOS: nitric oxide synthase; PG: prostaglandin; RT-PCR: reverse transcriptase quantitative polymerase chain reaction; TNF: tumor necrosis factor; WNT: wingless-type mouse mammary tumor virus (MMTV) integration site family.

## Competing interests

The authors declare that they have no competing interests.

## Authors' contributions

CF conceived the study, performed functional experiments, interpreted the data and drafted the manuscript. FMP performed RT-PCR experiments, interpreted the data and drafted the manuscript. MLG performed statistical analyses, interpreted the data and revised the manuscript. EN collected the tissue samples, interpreted the data and revised the manuscript. SGD performed ELISA analysis of organ-bath fluid and revised the manuscript. ES collected the tissue samples and revised the manuscript. MLC performed RT-PCR experiments, interpreted the data and revised the manuscript. PD participated in the design and coordination of the study and revised the manuscript. All authors read and approved the final manuscript.
